# Sensitivity of jarrah (*Eucalyptus marginata*) to phosphate, phosphite, and arsenate pulses as influenced by fungal symbiotic associations

**DOI:** 10.1007/s00572-015-0674-z

**Published:** 2016-01-26

**Authors:** Khalil Kariman, Susan J. Barker, Ricarda Jost, Patrick M. Finnegan, Mark Tibbett

**Affiliations:** School of Earth and Environment M087, The University of Western Australia, Crawley, WA 6009 Australia; School of Plant Biology M084, The University of Western Australia, Crawley, WA 6009 Australia; Institute of Agriculture M082, The University of Western Australia, Crawley, WA 6009 Australia; Centre for Agri-Environmental Research, and Soil Research Centre, School of Agriculture Policy and Development, University of Reading, Berkshire, RG6 6AR UK; School of Life Science, La Trobe University, Bundoora, VIC 3083 Australia

**Keywords:** *Eucalyptus marginata*, Ectomycorrhiza, Arbuscular mycorrhiza, Non-colonizing symbiosis, Toxicity, Phosphate/phosphite/arsenate, *PHT1* genes

## Abstract

Many plant species adapted to P-impoverished soils, including jarrah (*Eucalyptus marginata*), develop toxicity symptoms when exposed to high doses of phosphate (Pi) and its analogs such as phosphite (Phi) and arsenate (AsV). The present study was undertaken to investigate the effects of fungal symbionts *Scutellospora calospora*, *Scleroderma* sp., and *Austroboletus occidentalis* on the response of jarrah to highly toxic pulses (1.5 mmol kg^−1^ soil) of Pi, Phi, and AsV. *S. calospora* formed an arbuscular mycorrhizal (AM) symbiosis while both *Scleroderma* sp. and *A. occidentalis* established a non-colonizing symbiosis with jarrah plants. All these interactions significantly improved jarrah growth and Pi uptake under P-limiting conditions. The AM fungal colonization naturally declines in AM-eucalypt symbioses after 2–3 months; however, in the present study, the high Pi pulse inhibited the decline of AM fungal colonization in jarrah. Four weeks after exposure to the Pi pulse, plants inoculated with *S. calospora* had significantly lower toxicity symptoms compared to non-mycorrhizal (NM) plants, and all fungal treatments induced tolerance against Phi toxicity in jarrah. However, no tolerance was observed for AsV-treated plants even though all inoculated plants had significantly lower shoot As concentrations than the NM plants. The transcript profile of five jarrah high-affinity phosphate transporter (*PHT1* family) genes in roots was not altered in response to any of the fungal species tested. Interestingly, plants exposed to high Pi supplies for 1 day did not have reduced transcript levels for any of the five *PHT1* genes in roots, and transcript abundance of four *PHT1* genes actually increased. It is therefore suggested that jarrah, and perhaps other P-sensitive perennial species, respond positively to Pi available in the soil solution through increasing rather than decreasing the expression of selected *PHT1* genes. Furthermore, *Scleroderma* sp. can be considered as a fungus with dual functional capacity capable of forming both ectomycorrhizal and non-colonizing associations, where both pathways are always accompanied by evident growth and nutritional benefits.

## Introduction

Phosphorus (P) deficiency is common in many soils around the world including Australia, Sub-Saharan Africa, and tropical regions in Asia and South America (Sanchez and Buol 1975; Runge-Metzger 1995; Handreck 1997; Trolove et al. 2003; Smaling 2005). Adaptation to low P soils could potentially be linked to P sensitivity across native plant communities. Many Australian native species develop toxicity symptoms when exposed to high levels of inorganic phosphate (Pi) and its analogs such as phosphite (Phi) and arsenate (AsV) (Handreck 1997; Howard et al. 2000; Barrett 2001; Tynan et al. 2001; Smith et al. 2003; Shane et al. 2004a; Thomson and Leishman 2004; Hawkins et al. 2008; Pang et al. 2010a, b). According to Handreck (1997), an Olsen-extractable P concentration (inorganic plus organic P in the extract) of about 20 mg P kg^−1^ soil is lethal to the seedlings of P-sensitive native species. It has long been assumed that P-sensitive plants have lost their capacity to down-regulate the expression of their high-affinity phosphate transporter (*PHT1* family) genes in roots based on physiological observations (Shane et al. 2004b; Hawkins et al. 2008; Lambers et al. 2011), yet the molecular basis of this phenomenon has never been examined.

Plant roots take up P in the form of Pi through membrane-embedded PHT1 transporters. Some PHT1 transporters are located in the plasmalemma of plant root epidermal cells and root hair cells, such as MtPT1 in *Medicago truncatula* (Chiou et al. 2001) and StPT1 and StPT2 in potato (Rausch et al. 2001), and absorb Pi directly from the soil solution. A distinct subset of PHT1 transporters involved in the uptake of Pi released by arbuscular mycorrhiza (AM) hyphae within root cortical cells, and usually up-regulated following mycorrhizal colonization, include StPT3 and StPT4 in potato (Rausch et al. 2001), MtPT4 in *M. truncatula* (Harrison et al. 2002; Javot et al. 2007), and OsPT11 in rice (Paszkowski et al. 2002). As both groups are high-affinity PHT1 transporters, it is currently unclear what distinct purpose, if any, is held by the two subsets. It may be that the redundancy in PHT1 transporter function was required evolutionarily to enable distinct spatial patterns of expression in the presence and absence of an AM symbiosis. Mycorrhizal symbiosis may also cause the down-regulation or suppression of some *PHT1* transporter genes as the Pi uptake shifts from root epidermal to hyphal pathway in mycorrhizal roots. *MtPT1*, *MtPT2*, and *LePT1* are among *PHT1* transporter genes to be down-regulated during mycorrhizal colonization (Liu et al. 1998; Burleigh and Harrison 1999; Rosewarne et al. 1999; Chiou et al. 2001). Altered expression of *PHT1* transporter genes depending on their location within the plant root and also their involvement in direct (plant roots) or indirect (mycorrhizal) pathway of Pi uptake is reflective of the underlying importance of the AM symbiosis in plant nutrition genetics.

The main objective of the present research was to understand how the interactions that occur between plants and symbiotic fungi in the rhizosphere influence the nutritional status of plants that evolved in Pi-deficient conditions and in particular following exposure to toxic levels of Pi and its analogs Phi and AsV. Phosphate is the primary source of P that plants can take up. Phosphite, a more reduced form of phosphorus, is currently the only available treatment to effectively combat *Phytophthora cinnamomi* “dieback” in plants (Dell et al. 2005). It is generally considered to be a non-metabolizable form of P as it cannot be assimilated into organic P compounds or be oxidized to Pi by plants (Guest and Grant 1991). Therefore, application of Phi can result in the development of toxicity symptoms (Sukarno et al. 1993, 1996, 1998; Ticconi et al. 2001; Varadarajan et al. 2002). However, there are also reports on positive nutritional effects of Phi on plants (Jabahi-Hare and Kendrick 1987; Lovatt and Mikkelsen 2006), which are almost certainly due to microbe-mediated oxidation of Phi to Pi in soil (Ohtake et al. 1996). Arsenate and arsenite (AsIII) are the most common oxidation states of arsenic (As) found in nature. Contamination of groundwater and soil by As compounds is a result of natural processes such as the eruption of volcanoes and erosion of mineral deposits or anthropogenic activities such as mining, agriculture (application of P fertilizers laced with traces of As, arsenical pesticides, and herbicides), forestry, and drilling (Smith et al. 2003). In the cell, AsV can disturb respiratory energy production by substituting for P in the production of ATP, forming an ADP-As complex that uncouples ATP synthesis (Meharg and Hartley-Whitaker 2002; Finnegan and Chen 2012). In plants that are not colonized by root fungi, Phi and AsV appear to enter plant roots through the same mechanism as Pi, i.e., via Pi transporters of the PHT1 family located in the plasma membrane of epidermal and root hair cells (Meharg and Macnair 1992; Smith et al. 2000; Finnegan and Chen 2012).

The toxicity induced by Pi, Phi, or AsV might be affected by soil biological properties such as mycorrhizal associations. Arbuscular mycorrhizal, ectomycorrhizal (ECM), and non-colonizing (Kariman et al. 2014a) symbioses have been shown to induce tolerance in jarrah (*Eucalyptus marginata* Donn ex Sm.) seedlings against two consecutive toxic Pi pulses of 10 and 30 mg P kg^−1^ soil (Kariman et al. 2014b). Tolerance to mineral toxicity in mycorrhizal plants could be achieved through various mechanisms such as lower uptake, dilution in a higher plant mass, detoxification by fungal or plant metabolites, and sequestration of minerals within plant or fungal vacuoles (Hildebrandt et al. 2007). Plants colonized by AM (Karandashov and Bucher 2005; Javot et al. 2007) or ECM (Loth-Pereda et al. 2011; Kariman et al. 2014a) fungi have reduced transcript levels for some of their *PHT1* genes in roots. AM symbiosis has been shown to reduce AsV uptake in barley by suppressing the expression of the *PHT1* genes in roots that are involved in direct acquisition of Pi/As from soil (Christophersen et al. 2009). The mycorrhizal pathway, however, compensated the reduction in P uptake, which occurred due to down-regulation of root epidermal PHT1 transporters (Christophersen et al. 2009). Therefore, there might be a link between tolerance to Pi, Phi, and AsV toxicities in mycorrhizal plants and the expression of *PHT1* genes in roots.

Jarrah plants can form AM and ECM associations along with a recently described symbiosis involving Basidiomycete fungi, in which root colonization does not occur (Kariman et al. 2014a). ECM and non-colonizing symbioses can substantially improve eucalypt growth and nutrition (Jones et al. 1998; Chen et al. 2000; Kariman et al. 2012, 2014a, b). In the non-colonizing symbiosis with *A. occidentalis*, hyphae do not penetrate roots, and therefore, the fungal partner does not directly transfer nutrients to root cells, as commonly occurs in mycorrhizal symbioses. The improved nutrient uptake in plants harboring this novel symbiosis is linked to an enhanced carboxylate concentration in the rhizosphere soil (Kariman et al. 2014a). However, the AM-eucalypt symbiosis, which in nature appears restricted to the seedling state, is not always accompanied by growth and nutritional benefits (Gomez et al. 1987; Muchovej and Amorim 1990; Jones et al. 1998; Chen et al. 2000; Kariman et al. 2012, 2014b).

Experiments were conducted to investigate whether the toxic effects of Pi, Phi, and AsV on jarrah could be moderated by fungal symbionts. It was hypothesized that the expression of plant *PHT1* genes (as measured by transcript abundance) would be affected by symbiotic associations or toxicity. To answer these questions, jarrah plants were grown alone or in symbiosis with *S. calospora* (Nicol. & Gerd.) (AM), *Scleroderma* sp., or *A. occidentalis* (Watling & N.M. Greg.) (non-colonizing fungi) to (i) investigate the role of the selected fungi in inducing tolerance against highly toxic pulses of Pi, Phi, or AsV by monitoring both plant health and P or As uptake after short and long term exposures and (ii) quantify the transcript abundance of five *PHT1* genes in jarrah roots in response to different symbiotic associations or toxic pulses of Pi, Phi, or AsV.

## Materials and methods

### Plant materials, fungal isolates, and inoculum production

Seed capsules were obtained from a single jarrah tree near Dwellingup, Western Australia. Capsules were incubated at 42 °C for 3 days to release the seeds. The seeds were surface-sterilized with 70 % (*v*/*v*) ethanol for 1 min, rinsed with sterile water, and subsequently soaked in 4 % (*w*/*v*) sodium hypochlorite for 30 s. After rinsing with sterile water, seeds were germinated on moist filter paper in Petri dishes following incubation in the dark for 2 weeks at 15 °C. The three fungal isolates used were *S. calospora* (Nicol. & Gerd.) WUM 12 (3), *Scleroderma* sp., and *A. occidentalis* (Watling & N.M. Greg.). The AM inoculum (*S. calospora*) was bulked by growing leek plants in a mixture of AM inoculum and double-pasteurized washed river sand (1:9 *w*/*w*) for 4 months. The *A. occidentalis* isolate was collected at the Langford Park jarrah forest rehabilitation site (Jarrahdale, WA) from among cultivated *Eucalyptus resinifera* plants. The *Scleroderma* sp. isolate was collected from a Banksia woodland at Piney Lakes, WA.

The hyphal inocula for *A. occidentalis* or *Scleroderma* sp. treatments were prepared following a method adapted from Marx and Bryan (1975) using a vermiculite-based substrate. Medium grade vermiculite was mixed with Lithuanian peat moss (5:1 *v*/*v*), and 200 mL of the mixture was used to half-fill polyethylene jars followed by autoclaving. The contents of the jars were then moistened with 125 mL of liquid standard growth medium (Lambilliotte et al. 2004) and autoclaved again. After cooling down to room temperature, all jars were inoculated with 10 mycelial plugs (5 mm in diameter) taken from the edge of actively growing cultures on PDA plates and incubated at 23 °C for 2 months. Jarrah roots were stained using the ink and vinegar method (Vierheilig et al. 1998) with some modifications. Roots were cleared in 10 % (*w*/*v*) KOH at 90 °C for 1 h. After rinsing with water, roots were bleached in freshly made alkaline H_2_O_2_ [0.5 mL of 30 % (*v*/*v*) H_2_O_2_ and 0.5 ml of 28 % (*w*/*v*) NH_4_OH per 100-mL aqueous solution] for 30 min at room temperature. Roots were washed thoroughly to remove residual H_2_O_2_ and subsequently acidified by immersion in 10 % (*v*/*v*) HCl for 5 min before staining. To stain, roots were transferred to 5 % (*v*/*v*) black ink (Sheaffer) in white vinegar and incubated for 16 h. For destaining, roots were transferred to 5 % (*v*/*v*) white vinegar in deionized water for 30 min before storage in lactoglycerol (lactic acid, deionized water, and glycerol; 1:1:2).

The AM fungal colonization was measured using the gridline intersect method (Giovannetti and Mosse 1980), and more than 300 grid intersects were counted per sample.

### Inoculation and growth conditions

To prepare the AM treatment, viable *S. calospora* inoculum was mixed with double-pasteurized washed river sand (1:10 *w*/*w*) and subsequently mixed with sterilized *A. occidentalis* inoculum (10:1 *v*/*v*). Sterilized *A. occidentalis* inoculum was used here, as the same growth medium was used to produce both *A. occidentalis* and *Scleroderma* sp. inocula. To prepare *A. occidentalis* or *Scleroderma* sp. treatments, the respective inoculum was used along with the sterilized AM inoculum and mixed with double-pasteurized washed river sand using the same ratios as the AM treatment. Non-mycorrhizal plants also received sterilized AM and *A. occidentalis* inocula. This inoculation strategy was applied in order to have equal amounts of nutrients and organic matter in different treatments. Square plastic pots (8 × 8 × 18 cm) were lined with double plastic bags and filled with 1 kg of the growth substrate prepared as described above. Pre-germinated jarrah seeds (Kariman et al. 2012) were planted in pots containing the respective inoculum and double-pasteurized washed river sand as described above. The pot surface was covered with 3 cm of sterile plastic beads to minimize air-borne fungal contaminations. All plants received a modified Long Ashton solution lacking P once a fortnight commencing 2 weeks after planting (10 mL kg^−1^ substrate): 2 mM K_2_SO_4_, 1.5 mM MgSO_4_.7H_2_O, 3 mM CaCl_2_.2H_2_O, 0.1 mM FeEDTA, 4 mM (NH_4_)_2_SO_4_, 8 mM NaNO_3_, 46 μM H_3_BO_3_, 9 μM MnCl_2_.4H_2_O, 8 μM ZnSO_4_.7H_2_O, 0.3 μM CuSO_4_.5H_2_O, and 0.01 μM Na_2_MoO_4_.2H_2_O (Cavagnaro et al. 2001). Sealed pots were used for this experiment and watered to field capacity three times a week. The experiment was conducted from February to June 2012 in an unheated glasshouse with an average daytime temperature of 22 °C.

### Experimental design and treatments

Jarrah plants were grown alone or in presence of the three fungal isolates. Fourteen weeks after planting, three replicates from all non-mycorrhizal (NM) and inoculated treatments were harvested to analyze AM colonization and shoot P concentration of plants. The washed river sand used in this study contained less than 6 mg P kg^−1^ (data not shown). On the subsequent day after the first harvest, a toxic pulse (1.5 mmol kg^−1^ soil) of Pi (KH_2_PO_4_), Phi (KH_2_PO_3_), or AsV (Na_2_HAsO_4_) in aqueous solution was added to the respective treatments. To do this, 40 ml of solution containing 1.5 mmol of each chemical was evenly distributed over the surface of the substrate in the respective pots. Three replicates from each treatment were harvested 1 day after adding the pulses to monitor the P or As uptake during short-term exposure. The remaining plants were harvested four weeks after the pulse, apart from the AsV-treated plants which were harvested 1 week after the pulse due to the severity of symptoms. The root system was divided into three parts to be used for RNA isolation, colonization studies, and biomass measurements. The root subsamples taken for RNA isolation were immediately transferred to liquid nitrogen and subsequently stored at −80 °C. The root subsamples taken for colonization studies were transferred to plastic vials containing 50 % ethanol (*v*/*v*) until staining. Shoot and root samples taken for nutrient analysis and dry weight (DW) measurements were oven-dried at 70 °C for 72 h.

### Nutrient analysis and toxicity assessment

Measured quantities of ground-dried shoot tissues (about 200 mg) were digested in 5 ml nitric-perchloric acid solution (4:1 *v*/*v*). Total shoot P and As concentrations were determined using inductively coupled plasma optical emission spectrometry (ICP-OES; Optima 5300 DV, PerkinElmer, USA). Shoot-free Pi was also determined to clarify if the differential fractionation between organic P and Pi was correlated with Pi tolerance. A measured amount (about 40 mg) of dried ground tissues was homogenized in 1-mL acetic acid and used to measure the shoot-free Pi concentration following the ammonium molybdate method (Ames 1966) using the Multiskan Spectrum v1.2 plate reader (Thermo Electron Corporation, USA). The shoot organic P concentration was determined by calculating the difference between the total shoot P and free Pi concentrations.

Toxicity symptoms (including discolored, chlorotic, and necrotic areas on leaves) were quantified by ranking plants into six classes from 0 to 5, where 0 corresponded to the absence of toxicity symptoms; 1 ranged from traces to 20 % of symptomatic leaf tissue area (SLTA); 2 from 20 to 40 % SLTA; 3 from 40 to 60 % of SLTA; 4 from 60 to 80 % of SLTA; and 5 more than 80 % of SLTA.

### Phosphate transporter gene identification and relative transcript quantification

Complementary cDNAs from five *EmPHT1* genes were previously cloned and sequenced from jarrah roots (Kariman et al. 2014a). The GenBank accession numbers of the jarrah *PHT1* expressed sequence tags are as follows: *EmPHT1;1* (KC172372), *EmPHT1;2* (KC172373), *EmPHT1;3* (KC172374), *EmPHT1;4* (KC172375), and *EmPHT1;5* (KC172376). For transcript quantification, total root RNA was isolated using a CTAB-based method (Korimbocus et al. 2002) with a slight modification. Sodium D-isoascorbate was added to the extraction buffer just prior to use to a final concentration of 100 mM. Total RNA (1.0 μg) was treated with DNase I (RQ1 RNase-free DNase, Promega, USA) and subsequently reverse transcribed using the GoScript^™^ reverse transcriptase kit (Promega). The gene-specific primers EmPT1-F/-R, EmPT2-F/-R, EmPT5-F/-R, and Act2-F/-R (Table 1) were previously designed for three of the five jarrah PHT1 cDNAs and a jarrah actin sequence (*EmACT1*, KC172377) used as a reference for the transcript quantification assay (Kariman et al. 2014a). Here, primers EmPT3-F/-R and EmPT4-F/-R were designed for *EmPht1;3* and *EmPht1;4*, respectively (Table 1). SYBR Green-based quantitative real-time PCR (qPCR) was carried out to quantify the *PHT1* transcripts relative to *EmACT1* transcripts. A subsample of the cDNA synthesis reaction was removed prior to adding reverse transcriptase and included in qPCR assays to check for the presence of residual genomic DNA contamination in the RNA samples. qPCR reactions were performed in 96-well plates in a 10-μl reaction volume of 0.3 μM of each gene-specific primer and 2.5 μl of cDNA (synthesized using 50 ng total RNA) in 1× SYBR Green PCR master mix (Applied Biosystems, USA). All qPCR experiments were performed on a 7500 FAST Real-time PCR System (Applied Biosystems, USA). There were three biological replicates for this experiment.

**Table 1:** Gene-specific primers used for real-time PCR

Primer	Direction	Sequence (5′-3′)	Product size (bp)
EmPT1-F^a^	Forward	GAGCCGTCGAGATGGTGTGTAGA	122
EmPT1-R	Reverse	CGACTATCTTGCCACTTCCTCCATTGA	
EmPT2-F	Forward	CGATGAGGTGCCCACTGCT	138
EmPT2-R	Reverse	CACCTGCTCGACGACTCCGTAAT	
EmPT3-F	Forward	CAACAACTTCGGCTTGTTCAGCAGA	183
EmPT3-R	Reverse	ACTTCCTCAATTGCGTTCATGGTGTC	
EmPT4-F	Forward	TGGACATCGCCTTCTATAGCCAGAATCTT	113
EmPT4-R	Reverse	GCCCAATCCGGTACACCTCTTCTATG	
EmPT5-F	Forward	GGACGATGAGGTGTCCACTGCTT	127
EmPT5-R	Reverse	TCTGTAATATTCGGCAACACGGGAAGT	
Act2-F	Forward	GGTCCTGTTCCAACCATCCATGATT	136
Act2-R	Reverse	GGTAGAACCACCACTGAGGACAATGT	

^a^The sequences of primer pairs EmPT1, EmPT2, EmPT5, and Act2 (specific for *EmACT1*) were previously published (Kariman et al. 2014a)

### Cladistic analysis of PHT1 protein sequences

A cladogram was constructed to determine the hypothetical evolutionary relationship between jarrah PHT1 proteins and some PHT1 proteins associated with mycorrhizal interactions in other plant species. Yeast transporter sequences were used as an outgroup. Amino acid sequences deduced from *PHT1* cDNAs were aligned with Clustal Omega (Sievers et al. 2011). A neighbor-joining tree was constructed using 500 bootstrap replications and the Poisson model for amino acid substitutions (MEGA 6, Tamura et al. 2013).

### Statistical analyses

The experiment was conducted in a completely randomized design with three replicates at each time point. All data were analyzed using the Statistical Analysis System (SAS) version 9.2 (SAS Institute, Inc.; Cary NC, USA) software package. One-way ANOVAs were performed and means were separated using LSD at 5 % significance level. Two individual experiments were previously performed (Kariman et al. 2012, 2014a, b) using the same plant and fungal isolates and the same growth medium, where clear growth responses of jarrah to Pi deficiency/toxicity and the non-colonizing symbiosis were documented.

## Results

### Plant mass before pulse addition

**Fig. 1:**
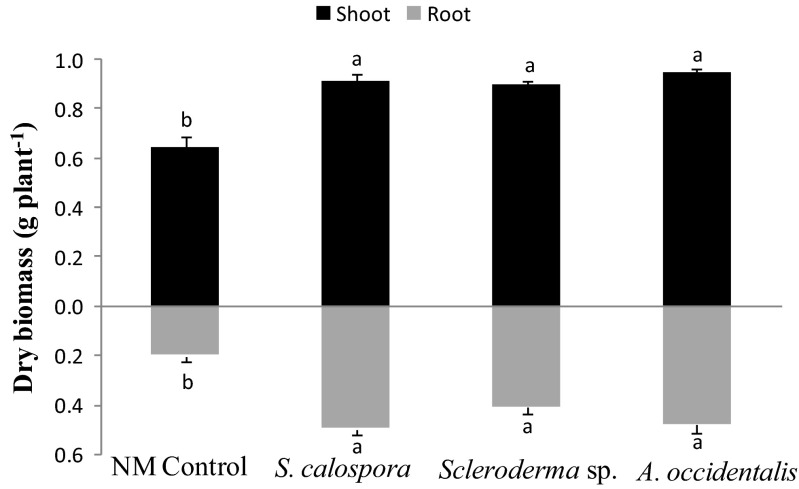
Root and shoot dry mass of inoculated and uninoculated (NM) jarrah plants after 14 weeks of growth under P-limiting conditions. *Bars* labelled with different letters are significantly different at *p* < 0.05. *Error bars* are SE (*n* = 3)

Jarrah seedlings inoculated with any one of the three fungal species had significantly higher root and shoot dry mass compared to NM plants after 14 weeks of growth under P-limiting conditions (Fig. 1). There was no significant difference between the root and shoot mass among different inoculated treatments.

### Mycorrhizal colonization

**Fig. 2:**
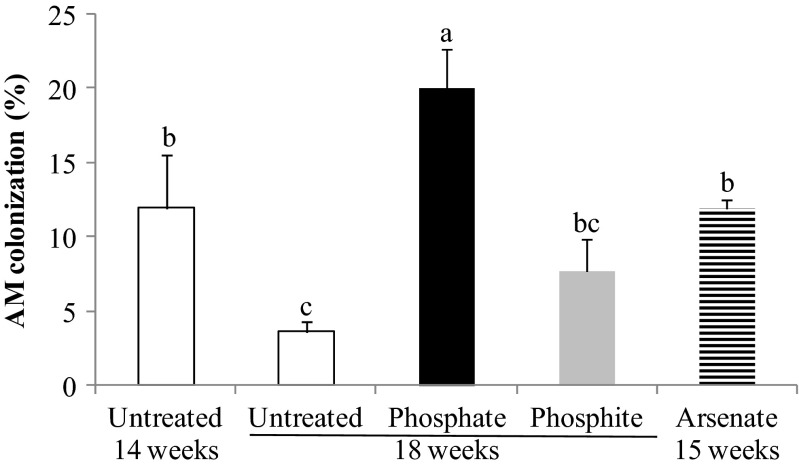
Percentage of mycorrhizal colonization in the AM treatment (*S. calospora*). The root colonization was measured in 14- and 18-week-old P-limited plants and also for 18-week-old plants which were exposed to Pi (*closed bars*) or Phi (*grey bars*) pulses for 4 weeks or 15-week-old plants that had been exposed to the AsV pulse (*striped bars*) for 1 week. *Bars* labelled with different letters are significantly different at *p* < 0.05. *Error bars* are SE (*n* = 3)

No root colonization was observed for *Scleroderma* sp. and *A. occidentalis* treatments although positive growth and P nutritional effects were evident, indicating that both fungi had established a non-colonizing symbiosis with jarrah. For the AM fungus *S. calospora*, there was 12 % root colonization after 14 weeks growth (start of the pulse experiment). Four weeks after the treatments, colonization was reduced to 3 % in untreated plants (Fig. 2). However, in plants that received the toxic Pi pulse, the AM colonization was significantly higher than in both 14- and 18-week-old untreated plants. The AsV-treated plants were harvested 7 days after adding the pulse, and root colonization was unchanged from that of untreated AM plants at the beginning of the experiment (12 %). There was no significant difference between AM colonization of Phi-treated plants, 14-week-old untreated plants at the beginning of the experiment, and 18-week-old untreated plants.

### Tolerance to phosphate, phosphite and arsenate toxicities

**Fig. 3:**
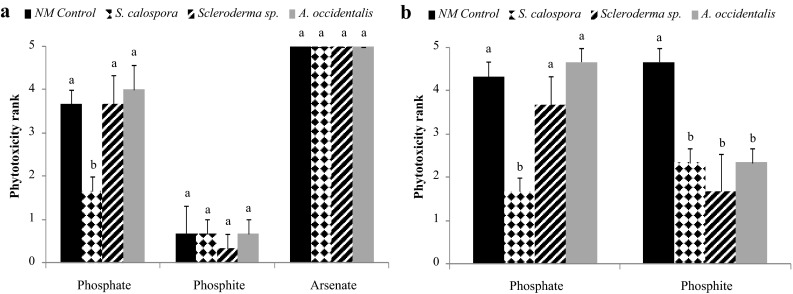
Toxicity ranks of jarrah plants exposed to toxic pulses of Pi, Phi, and AsV 1 week (**a**) and 4 weeks (**b**) after the pulse. There were no toxicity symptoms in untreated jarrah seedlings. For each treatment, *bars* labelled with different letters are significantly different at *p* < 0.05. *Error bars* are SE (*n* = 3)

At the end of experiment, there were no toxicity symptoms in the set of NM seedlings that were not exposed to Pi, Phi, or AsV pulses. Tolerance to a normally toxic pulse of Pi was only observed for the AM plants, which had significantly lower toxicity rankings compared to NM plants, both 1 and 4 weeks after adding the Pi pulse (Fig. 3a, b). When a toxic pulse of Phi was applied, slight toxicity symptoms were observed in all treatments 1 week after the pulse. At that point, the toxicity ranks did not differ significantly between inoculated and control treatments. Four weeks after the Phi pulse, however, all inoculated plants had significantly lower toxicity symptoms compared to NM plants. There was no tolerance against the AsV toxicity, regardless of symbiotic status, and all plants had died within a week after the pulse.

### Shoot mass of plants under phosphate, phosphite, or arsenate treatment

**Fig. 4:**
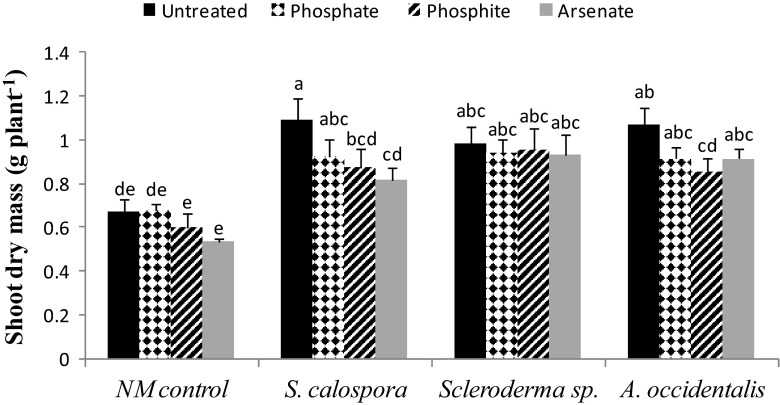
Shoot dry mass of 18-week-old jarrah plants grown under P-limiting conditions (controls, *closed bars*), 18-week-old plants which were exposed to Pi (*checkered bars*) or Phi (*striped bars*) pulses for 4 weeks or 15-week-old plants that had been exposed to AsV (*grey bars*) for 1 week. *Bars* with different letters are significantly different at *p* < 0.05. *Error bars* are SE (*n* = 3)

There was a significant reduction in shoot mass of Phi-treated plants in the *A. occidentalis* treatment (21 %) and of Phi (19 %) or AsV-treated plants (26 %) in the *S. calospora* treatment, compared to the respective untreated plants (Fig. 4). Other than these, there was a slight decrease in shoot dry mass of inoculated plants treated by Pi (<16 %), Phi (<11 %), or AsV (<15 %) pulses compared to their respective untreated controls; however, the differences were not statistically significant.

### Shoot P concentration and content in the phosphate-treated plants

**Fig. 5:**
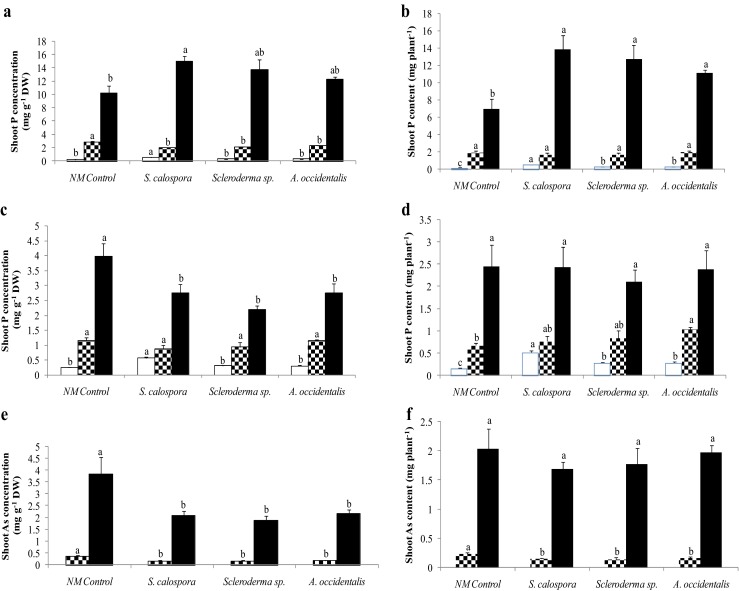
Phosphorus and As uptake of NM and inoculated jarrah plants. **a**, **b** Shoot P concentration and content of plants 1 day before (*open bars*), 1 day after (*checkered bars*), and 4 weeks after (*closed bar*) the Pi pulse. **c**, **d** Shoot P concentration and content 1 day before (*open bars*), 1 day after (*checkered bars*), and 4 weeks after (*closed bars*) the Phi pulse. **e**, **f** Shoot As concentration and content 1 day (*checkered bars*) and 1 week (*closed bars*) following exposure to the AsV pulse. *Bars* of each type labelled with different letters are significantly different at *p* < 0.05. *Error bars* are SE (*n* = 3)

One day prior to exposure to the toxic Pi pulse, AM plants had a significantly higher shoot P concentration compared with other treatments (Fig. 5a, open bars). One day after the pulse, all inoculated plants had significantly lower shoot P concentration than NM plants (Fig. 5a, checkered bars). Four weeks after the Pi pulse, AM plants had significantly higher shoot P concentration than did NM plants (Fig. 5a, closed bars), while there was no significant difference in shoot P concentration between both *Scleroderma* sp. and *A. occidentalis* treatments and NM plants (Fig. 5a, closed bars).

Due to a positive growth response, the three fungal species could enhance the shoot P content of plants under P-limiting conditions compared to NM plants prior to the treatments (Fig. 5b, open bars). A day after the Pi pulse, the shoot P content did not differ significantly across treatments (Fig. 5b, checkered bars). All inoculated plants had significantly higher shoot P content than did NM plants 4 weeks after adding the Pi pulse (Fig. 5b, closed bars).

### Shoot P concentration and content in the phosphite-treated plants

There was no significant difference between the shoot P concentrations among treatments 1 day after the Phi pulse (Fig. 5c, checkered bars). Four weeks after the pulse, all inoculated plants had significantly lower shoot P concentrations than did NM plants (Fig. 5c, closed bars). Plants inoculated with *A. occidentalis* had significantly higher shoot P content than did NM plants after 1 day of exposure to the Phi pulse (Fig. 5d, checkered bars), while *S. calospora* and *Scleroderma* sp. treatments had slightly (not significant) higher shoot P content than did NM plants. After 4-week exposure to the pulse, the shoot P content did not differ significantly across treatments (Fig. 5d, closed bars).

### Shoot As concentration and content in the arsenate-treated plants

All inoculated plants had significantly lower shoot As concentrations compared to NM plants 1 day and 1 week after addition of the AsV pulse (Fig. 5e). Inoculated plants also had lower shoot As content 1 day after adding the pulse compared to NM plants. A week after the pulse, the shoot As content did not differ significantly across treatments (Fig. 5f). However, all plants were dead 1 week after exposure to the AsV pulse.

### Shoot free Pi concentration

**Fig. 6:**
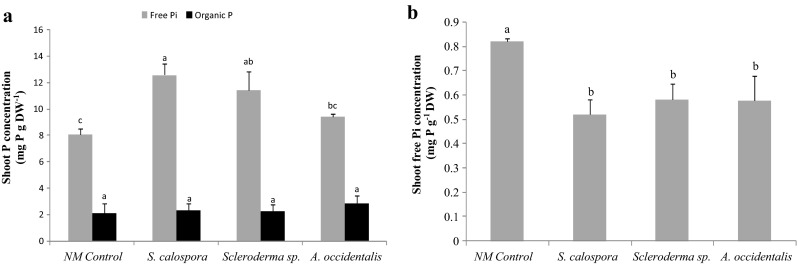
Shoot Pi and organic P concentration in jarrah plants after 4-week exposure to toxic pulses of Pi or Phi. **a** Concentration of Pi (*grey bars*) and organic P (*closed bars*) after the Pi pulse. **b** Concentration of shoot Pi after the Phi pulse. *Bars* of each type labelled with different letters are significantly different at *p* < 0.05. Error bars are SE (*n* = 3)

To determine the fractionation between free Pi and organic P pools inside the plants, the shoot Pi concentration was measured (Fig. 6). Consistent with the total shoot P results (Fig. 5a), the AM plants had the highest free Pi concentration in their shoot tissues 4 weeks after the Pi pulse (Fig. 6a, grey bars). The shoot free Pi concentration in the *Scleroderma sp.* treatment was also significantly higher than in NM plants, while the *A. occidentalis* treatment had nearly the same free Pi as the NM plants. There was no significant difference between shoot organic P concentration across treatments as determined by the difference between total shoot P and free Pi concentrations (Fig. 6a, closed bars). Four weeks after the Phi pulse, NM plants had the highest shoot free Pi concentration (Fig. 6b), similar to the total shoot P concentrations (Fig. 5c).

### Response of *EmPHT1* transcript abundance to fungal symbionts or toxicity conditions

**Fig. 7:**
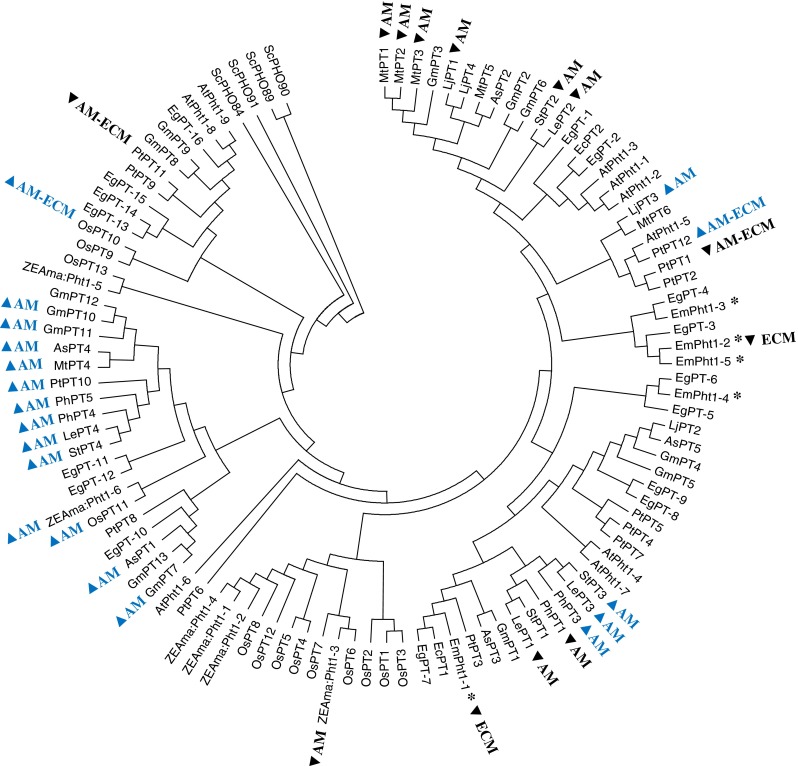
Cladogram constructed for PHT1 amino acid sequences from jarrah and other plant species. Transporters labelled with *inverted triangle* (in black) or  (in blue) have been shown to be down-regulated or induced by the indicated symbioses, respectively. The plant species and corresponding PHT1 sequences are: jarrah, *EmPHT1* (starred, Kariman et al. 2014a); *Eucalyptus camaldulensis*, *EcPT* (Koyama et al. 2006); poplar, *PtPT* (Loth-Pereda et al. 2011); tomato, LePT (Daram et al. 1998; Rosewarne et al. 1999; Nagy et al. 2005, 2009; Chen et al. 2014); potato StPT (Rausch et al. 2001; Nagy et al. 2005); *Medicago truncatula*, MtPT (Liu et al. 1998; Harrison et al. 2002; Grunwald et al. 2009); *Zea mays*, *ZmPT* (Tian et al. 2013); rice, OsPT (Paszkowski et al. 2002); *Lotus japonicus*, LjPT (Maeda et al. 2006; Deguchi et al. 2007); soybean, GmPT (Tamura et al. 2012); *Astragalus sinicus*, AsPT (Xie et al. 2013); *Petunia hybrida*, PhPT (Wegmüller et al. 2008; Breuillin et al. 2010); *Arabidopsis thaliana*, *AtPht1* (Mudge et al. 2002) and *Eucalyptus grandis*, EgPT (Myburg et al. 2014). The *E. garandis* putative *PHT1* genes were extracted from the Phytozome database and their transcript names are as follows: EgPT1 (Eucgr.H03064.1), EgPT2 (Eucgr.H00165.1), EgPT3 (Eucgr.H03067.1), EgPT4 (Eucgr.H03069.1), EgPT5 (Eucgr.H00161.1), EgPT6 (Eucgr.H00162.1), EgPT7 (Eucgr.H03062.1), EgPT8 (Eucgr.B01557.1), EgPT9 (Eucgr.K03265.1), EgPT10 (Eucgr.J00101.1), EgPT11 (Eucgr.A02668.1), EgPT12 (Eucgr.K00323.1), EgPT13 (Eucgr.F01808.1), EgPT14 (Eucgr.F01809.1), EgPT15 (Eucgr.F01811.1), EgPT16 (Eucgr.F03590.1). Phosphate transporter sequences from yeast *Saccharomyces cerevisiae* (ScPHO) were also included as outgroup sequences (Wykoff and O’Shea 2001)

The cladistic analysis suggested that all five jarrah PHT1 protein sequences are likely to be more closely related to those isoforms from other plant species that are involved in direct Pi uptake from soil and that are down-regulated in mycorrhizal roots (Fig. 7). Also, proteins encoded by most mycorrhiza-inducible *PHT1* genes fell within one cluster separate from the *EmPHT1* genes studied here.

**Fig. 8:**
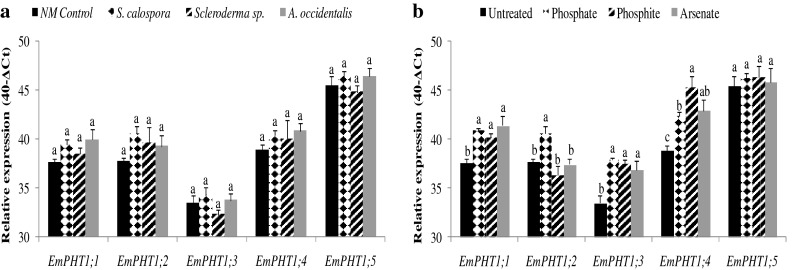
Transcript abundance from five *EmPHT1* genes in jarrah roots relative to an internal reference gene (*EmACT1*) determined by real-time PCR. **a** Transcript abundance from *EmPHT1* genes in response to the three different fungal species. **b** Transcript abundance from *EmPHT1* genes in NM plants under normal conditions (*closed bars*) or 1 day after addition of Pi (*checkered bars*), Phi (*striped bars*), or AsV (*grey bars*) pulses. The scale on the vertical axis is a log2 scale based on ΔCt (the threshold cycle (Ct) of the target *EmPHT1* gene minus the Ct of the reference gene). Thus a difference of one C_T_ value corresponds to a twofold difference in transcript abundance. The ∆C_T_ value is subtracted from 40, maximum number of PCR cycles, to make all values positive for ease of comparison (Bari et al. 2006). *Bars* with different letters are significantly different at *p* < 0.05. *Error bars* are SE (*n* = 3)

The transcript abundance from five jarrah *EmPHT1* genes was quantified in 14-week-old plants prior to addition of the toxic pulses (Fig. 8a). Jarrah plants did not have transcript abundance for these five *EmPHT1* genes in response to any of the fungal inoculations. In addition, the transcript abundance of these five *EmPHT1* genes in NM plants 1 day after addition of the different toxic pulses (Fig. 8b) was not reduced in response to the addition of Pi, Phi, or AsV compared to untreated plants. On the contrary, abundance of transcripts from several *EmPHT1* genes was increased under toxicity conditions. Jarrah plants accumulated significantly more transcripts for *EmPHT1;1*, *EmPHT1;2*, *EmPHT1;3*, and *EmPHT1;4* in response to Pi and for *EmPHT1;1*, *EmPHT1;3*, and *EmPHT1;4* in response to Phi or AsV pulses. The *EmPHT1;5* transcript level remained unchanged after the Pi, Phi, or AsV treatments.

## Discussion

As previously observed by Kariman et al. (2012, 2014a), the *A. occidentalis* isolate established a non-colonizing symbiotic association with jarrah plants and a growth benefit occurred without root colonization. *Scleroderma* sp. responded like *A. occidentalis* and did not form mycorrhizal structures in any of the jarrah plants. In an earlier study, *Scleroderma* sp. formed a classic ECM colonization in one replicate jarrah plant, while it did not colonize roots in other replicates and behaved like *A. occidentalis* (Kariman et al. 2012). Also, *Scleroderma* sp. formed ECM with jarrah in the study by Kariman et al. (2014a) and in a preliminary experiment (data not shown). These two fungi have always provided clear growth and nutritional benefits regardless of their colonization ability (Kariman et al. 2012, 2014a) suggesting that *Scleroderma* sp. is a fungus with dual functional capacity, sometimes behaving like a typical ECM fungus and sometimes establishing a non-colonizing symbiotic association like *A. occidentalis*. The present experiments were carried out in an unheated glasshouse and nighttime temperature dropped down to 6 and 7 °C in the study by Kariman et al. (2012) where ECM colonization by *Scleroderma* sp. was observed only in one replicate plant. However, in the study by Kariman et al. (2014a) and in the abovementioned preliminary experiment, *Scleroderma* sp. formed ECM colonization in all replicate jarrah plants, minimum night temperatures were 14 and 18 °C, respectively. Hence, there might be a relationship between temperature and the dual behavior of *Scleroderma* sp. A temperature drop to below 10 °C could be a potential signal for the fungus to establish a non-colonizing symbiotic association. Regardless of what drives this dual behavior, the colonization results from the current and previous experiments revealed that the non-colonizing symbiosis is an alternative pathway for certain ECM fungi to provide a benefit to the host plant. In other words, rhizosphere-inhabiting fungi such as *Scleroderma* sp. have the potential to establish both ECM and non-colonizing symbiotic associations during their life cycle.

The AM colonization of jarrah plants by *S. calospora* declined after 14 weeks of growth. This is in line with the existing literature about AM-eucalypt symbioses showing that AM colonization decreases after seedlings have grown for a while and is replaced by ECM colonization (Chen et al. 2000; Adams et al. 2006). Addition of Pi does not have a uniform effect. It often leads to a decrease in mycorrhizal colonization (Jasper and Davy 1993; Bobbink 1998; Cornwell et al. 2001), but other studies showed that AM colonization was enhanced (Bolan et al. 1984) or not affected by higher Pi supply (Duke et al. 1994; Kabir et al. 1997). In the present study, jarrah plants exposed to the Pi pulse for 4 weeks had significantly higher AM colonization than did both 14- and 18-week-old untreated plants. Here, the P availability in the substrate (less than 6 mg P kg^−1^) was very limited and addition of the Pi pulse apparently extended the mutualism for a longer period. Accordingly, it is hypothesized that AM relationships are not suppressed in 3-month-old jarrah eucalypt seedlings if they receive high doses of Pi. It would be interesting to test if ECM symbiosis can replace AM symbiosis under high Pi conditions.

The Phi-treated jarrah plants had slightly (not significant) higher AM colonization than did 18-week-old AM plants grown under P-limiting conditions. Just as with added Pi, inconsistent results have been reported for the effects of Phi treatment on AM colonization. An increase in AM colonization has been reported for plant species such as *Agonis flexuosa* (Howard et al. 2000), leek (Jabahi-Hare and Kendrick 1987), and lettuce (Clarke 1978) after Phi application. The increased AM colonization in Phi-treated plants has been attributed to differences in host nutritional uptake or altered root metabolism (Jabahi-Hare and Kendrick 1987; Howard et al. 2000).

All inoculated jarrah plants had significantly higher shoot P content than did NM plants under P-limiting conditions. The improved P nutrition of the plants which established a non-colonizing symbiosis with *Scleroderma* sp. or *A. occidentalis* could be linked to enhanced concentration of carboxylates in the rhizosphere soil leading to higher Pi availability for roots (Kariman et al. 2014a). In the study of Kariman et al. (2012), the AM (*S. calospora*) jarrah plants had slightly higher shoot P content than did NM plants although the difference was not statistically significant. Here, AM plants responded extraordinarily well compared to the previous study (Kariman et al. 2012), by having the highest shoot P content among treatments. The transcript abundance of jarrah *PHT1* genes might clarify the high performance of *S. calospora* in terms of Pi uptake as roots colonized with this AM fungus did not show reduced expression of any of the *EmPHT1* genes. Therefore, the shoot P content of the AM plants might have come from both direct (root epidermal) and indirect (mycorrhizal) pathways, but the actual contribution of each pathway is not clear and warrants additional study.

The Pi pulse applied of 1.5 mmol kg^−1^ soil is equivalent to 46 mg P kg^−1^ soil. Pi tolerance was only observed for the AM treatment, and all inoculated plants in the present study had significantly lower shoot P concentration than did NM plants 1 day after the Pi pulse. This was related to the higher shoot mass of inoculated plants, resulting in P dilution within shoot tissues. Interestingly, at the end of the experiment, AM plants had the highest shoot P concentration (about 15 mg P g^−1^ DW) and still showed the lowest toxicity symptoms among inoculated and NM plants. Pi tolerance in AM jarrah plants is therefore not necessarily correlated with lower shoot P concentration and seems to depend on the AM isolate involved. As previously reported, the AM fungus *Rhizophagus irregularis* conferred tolerance to jarrah against Pi toxicity by significantly reducing the shoot P concentration, whereas tolerance in the *S. calospora* treatment was accompanied by a slight (not significant) reduction in shoot P concentration compared to NM plants (Kariman et al. 2014b). One possible mechanism for the induced Pi tolerance could be that AM plants have the ability to assimilate inorganic P into organic pools and therefore do not accumulate free Pi, which causes toxicity symptoms. The shoot free Pi concentration of plants was measured to test this hypothesis. As seen before for total shoot P concentration, AM plants also had the highest shoot Pi concentration among inoculated and NM plants. The difference between total shoot P and free Pi (organic P) was less than 3 mg P g^−1^ DW and remained unchanged across treatments. This indicates that jarrah is not very efficient in converting Pi into organic P compounds. Therefore, the Pi tolerance in the AM jarrah plants is not linked with the plants’ ability to more quickly assimilate inorganic P into organic P pools. Activation of plant defense mechanisms could also be a possible explanation for the tolerance observed against Pi or Phi pulses; however, further research is required to understand this phenomenon. Plant species have been shown to reduce the expression of some of their *PHT1* genes in response to AM (Karandashov and Bucher 2005; Javot et al. 2007) and ECM (Loth-Pereda et al. 2011; Kariman et al. 2014a) colonization. This may not be the mechanism for the induced Pi tolerance in jarrah plants inoculated with the AM fungus *S. calospora* as a reduction in transcript abundance was not observed for any of the five *PHT1* genes tested. However, the genome of jarrah has not been sequenced yet and there might be additional *PHT1* genes that might negatively respond to high Pi conditions.

The other two fungal treatments (*Scleroderma* sp. and *A. occidentalis*) did not induce Pi tolerance in the present study. However, these fungi were able to induce tolerance in jarrah plants exposed to lower P pulses (two consecutive P pulses of 10 and 30 mg P kg soil^−1^) (Kariman et al. 2014b), probably because the inoculated plant shoot mass was higher and so their shoot P concentrations (less than 7.5 mg P g^−1^ DW) were about 20 % lower than those of NM plants. Here, at the time of pulse addition, the shoot mass of plants was relatively lower than that of plants in the previous experiment, plants received a higher pulse at once (46 mg P kg^−1^ soil), and the shoot P concentration was more than 12 mg P g^−1^ DW. It is thus concluded that the Pi tolerance in both *Scleroderma* sp. and *A. occidentalis* treatments that was previously reported (Kariman et al. 2014b) may be due to dilution effects by growth, magnitude of the P pulses, and the initial shoot size of the inoculated plants. Moreover, all the inoculated plants in this experiment had significantly higher shoot P content than did NM plants 4 weeks after the Pi pulse indicating that these fungi did not cause jarrah plants to reduce their net Pi uptake under P-toxic conditions.

From a plant perspective, Phi is a non-metabolizable form of P (Guest and Grant 1991) and the positive nutritional effects of Phi on plants (Jabahi-Hare and Kendrick 1987; Lovatt and Mikkelsen 2006) are most likely due to microbial oxidation of Phi to Pi in soil (Adams and Conrad 1953; Ohtake et al. 1996; White and Metcalf 2007) and/or suppression of plant diseases (Thao and Yamakawa 2009). Here, slight toxicity symptoms were present in all plants a week after the Phi pulse, and the shoot P concentrations did not differ significantly between treatments. Four weeks after the pulse, the shoot P concentration of jarrah plants was significantly lower in all the fungal treatments than the NM plants, which correlates with the reduced toxicity symptoms observed in all inoculated plants. At the final harvest, there was no significant difference between the shoot P content across treatments between inoculated or NM plants. This means that inoculated plants with higher shoot mass had the same shoot P content as NM plants indicating similar Phi uptake rates between them. Interestingly, all inoculated plants had significantly higher shoot P content than did NM plants 4 weeks after the Pi pulse. This suggests that the differentiation between Phi (a toxic P source) and Pi (metabolizable P source) is much stronger in AM and non-colonizing symbioses compared with NM plants, i.e., the symbiotic plants seem to preferentially take up Pi.

The shoot free Pi concentration was also determined in plants exposed to the Phi pulse for 4 weeks. This was done to clarify if symbiotic fungi can oxidize Phi to Pi in the soil as a potential mechanism for the Phi tolerance observed. The results did not support this hypothesis as NM plants had the highest shoot free Pi concentration similar to the total shoot P results. This might mean that these fungal species are not involved in oxidation of Phi to Pi in the soil. Phi can be taken up directly by roots (Darcylameta and Bompeix 1991). That was not investigated in this work but is the apparent reason for the toxicity symptoms observed.

Mycorrhizal fungi can induce tolerance against AsV toxicity by mechanisms including the reduction of AsV to AsIII, binding of phytochelatins to AsIII, accumulation of these peptide-metal complexes inside fungal vacuoles, and the down-regulation of plant high-affinity *PHT1* genes (Meharg and Macnair 1992; Sharples et al. 2000; Hildebrandt et al. 2007). Although the induced tolerance to As toxicity often improves growth and Pi uptake (Ahmed et al. 2006; Xu et al. 2008), As tolerance has also been observed where AM symbioses do not enhance growth or Pi nutrition of host plants (Christophersen et al. 2009). In the present study, no tolerance was recorded against AsV toxicity in terms of reducing the toxicity symptoms and all plants were dead a week after adding the pulse. The average As toxicity threshold for crop plants is about 40 μg g^−1^ DW (Sheppard et al. 1992), which is far below the concentrations observed in the present experiment and the apparent reason for death of all AsV-treated plants. In the short-term, however, different fungal treatments were able to reduce the shoot As concentration under AsV toxicity conditions. One day after adding the AsV pulse, inoculated plants had lower shoot As content compared with NM plants. It seems that inoculated plants reduced their net AsV uptake similar to what was observed for Phi, the other toxic Pi analog, as they had higher shoot mass but nearly the same shoot As content as NM plants. Here, reduced transcript abundance was not observed for any of the plant *PHT1* genes tested under symbiotic associations.

The abundance of transcripts from five jarrah *EmPHT1* genes was quantified by real-time PCR in roots of 14-week-old plants prior to addition of the different pulses to determine if there was a correlation with symbiotic associations. Five *PHT1* genes have been identified in *E. marginata* (Kariman et al. 2014a), although there are likely to be additional genes, based on the size of the *PHT1* gene family in other plants (Mudge et al. 2002; Paszkowski et al. 2002; Glassop et al. 2007; Loth-Pereda et al. 2011). Jarrah plants did not have reduced expression of the identified *PHT1* genes in roots in response to any of the fungal species tested, which were representatives of non-colonizing and AM symbioses. The expression profile of *PHT1* genes was also not altered in roots of plants harboring the non-colonizing symbiosis in a previous study (Kariman et al. 2014a). Recently, Loth-Pereda et al. (2011) showed that poplar plants reduce the expression of two *PHT1* genes in response to both AM and ECM symbioses. In a previous study, *Scleroderma* sp. behaved like a typical ECM fungus having a high colonization rate of 77 %. In this case, two *EmPHT1* genes (*EmPHT1;1* and *EmPHT1;2*) had significantly lower transcript levels in ECM roots than in NM roots (Kariman et al. 2014a). In the present study, *Scleroderma* sp. established a non-colonizing symbiotic interaction with jarrah and this difference may explain why the transcript abundance of those *PHT1* genes was not affected.

Jarrah plants did not have reduced *PHT1* gene transcript levels in response to the AM fungus *S. calospora*. Many plant species have reduced expression of select *PHT1* genes in response to AM colonization (Karandashov and Bucher 2005; Javot et al. 2007). The lack of change in the expression of *PHT1* genes in AM jarrah roots could be related to either the genetic characteristics of jarrah as a low-P adapted species or the transitory nature of the AM-eucalypt symbioses (Chen et al. 2000; Adams et al. 2006). The *PHT1* gene family in plants typically has more than five members, including nine genes in *Arabidopsis thaliana* (Mudge et al. 2002), 13 genes in *Oryza sativa* (Paszkowski et al. 2002; Glassop et al. 2007), and 12 genes in *Populus trichocarpa* (Loth-Pereda et al. 2011). None of the five *PHT1* gene sequences identified in jarrah to date cluster with those that have been shown to respond positively to mycorrhizal colonization in other species. Therefore, it is likely that yet-to-be identified *PHT1* gene members in jarrah roots will be found that respond to AM symbiosis.

Plant species that are not P-sensitive are very quick to reduce the expression of *PHT1* genes in their roots in response to high P supply (Burleigh and Harrison 1999; Rausch and Bucher 2002; Grunwald et al. 2009). Conversely, none of the jarrah *PHT1* genes examined were reduced in their expression 1 day after treatment with Pi or one of its two chemical analogs. In fact, the transcript abundance of four *PHT1* genes increased under Pi toxicity conditions. The enhanced transcript abundance of *PHT1* genes could have occurred (i) due to a positive response to the availability of Pi in a species adapted to a low-P environment and/or (ii) to facilitate the internal translocation of these additional target molecules inside plant tissues. It is therefore suggested that jarrah, and perhaps other P-sensitive perennial species, regulate the expression of at least some of their *PHT1* genes in response to the external P concentration (soil) rather than the plant internal P status. This would be the complete opposite to most plant species that induce *PHT1* genes in response to a lack of Pi availability and a low internal P status, but repress *PHT1* genes as soon as Pi becomes more readily available. Interestingly, a stimulating effect of Pi on *PHT1* expression has also been observed in roots of a P-efficient wheat cultivar (Aziz et al. 2014). In *Arabidopsis*, all *PHT1* genes are down-regulated in response to increased Pi supply, but the kinetics of the down-regulation differ among family members (Lapis-Gaza et al. 2014).

The results of the present study demonstrate that the fungal symbionts tested, regardless of their root colonization ability, can substantially improve jarrah growth and P nutrition under P-limiting conditions and potentially protect plants subjected to toxic levels of Pi or Phi. In the case of As, inoculated plants had significantly lower As concentration than did non-inoculated plants 1 week after the AsV pulse but the difference in As concentration did not allow protection of plants, and the shoot As content was the same across treatments. The enhanced transcript abundance of four *PHT1* genes in jarrah roots (including those shown to be down-regulated in ECM roots) under high Pi conditions suggest that expression of at least some *PHT1* genes is regulated by soil P availability rather than the actual plant P status. It would be interesting to further clarify if adequate or even toxic supplies of Pi can extend the duration of symbiotic relationships between eucalypts and AM fungi, which decline naturally after 2–3 months but were prolonged in this study following Pi application.
